# Genomic investigation of *Staphylococcus aureus* recovered from Gambian women and newborns following an oral dose of intra-partum azithromycin

**DOI:** 10.1093/jac/dkz341

**Published:** 2019-08-19

**Authors:** Abdoulie Bojang, Sarah L Baines, Liam Donovan, Romain Guerillot, Kerrie Stevens, Charlie Higgs, Christian Bottomley, Ousman Secka, Mark B Schultz, Anders Gonçalves da Silva, Torsten Seemann, Timothy P Stinear, Anna Roca, Benjamin P Howden

**Affiliations:** 1 Medical Research Council Unit The Gambia at the London School of Hygiene and Tropical Medicine, Fajara, The Gambia; 2 Doherty Applied Microbial Genomics, Department of Microbiology & Immunology, The University of Melbourne at The Peter Doherty Institute for Infection & Immunity, Melbourne, Victoria, Australia; 3 Microbiological Diagnostic Unit Public Health Laboratory, Department of Microbiology & Immunology, The University of Melbourne at The Peter Doherty Institute for Infection & Immunity, Melbourne, Victoria, Australia; 4 Medical Research Council Tropical Epidemiology Group, London School of Hygiene and Tropical Medicine, London, UK

## Abstract

**Background:**

Oral azithromycin given during labour reduces carriage of bacteria responsible for neonatal sepsis, including *Staphylococcus aureus*. However, there is concern that this may promote drug resistance.

**Objectives:**

Here, we combine genomic and epidemiological data on *S. aureus* isolated from mothers and babies in a randomized intra-partum azithromycin trial (PregnAnZI) to describe bacterial population dynamics and resistance mechanisms.

**Methods:**

Participants from both arms of the trial, who carried *S. aureus* in day 3 and day 28 samples post-intervention, were included. Sixty-six *S. aureus* isolates (from 7 mothers and 10 babies) underwent comparative genome analyses and the data were then combined with epidemiological data. Trial registration (main trial): ClinicalTrials.gov Identifier NCT01800942.

**Results:**

Seven *S. aureus* STs were identified, with ST5 dominant (*n* = 40, 61.0%), followed by ST15 (*n* = 11, 17.0%). ST5 predominated in the placebo arm (73.0% versus 49.0%, *P* = 0.039) and ST15 in the azithromycin arm (27.0% versus 6.0%, *P* = 0.022). In azithromycin-resistant isolates, *msr*(A) was the main macrolide resistance gene (*n* = 36, 80%). Ten study participants, from both trial arms, acquired azithromycin-resistant *S. aureus* after initially harbouring a susceptible isolate. In nine (90%) of these cases, the acquired clone was an *msr*(A)-containing ST5 *S. aureus*. Long-read sequencing demonstrated that in ST5, *msr*(A) was found on an MDR plasmid.

**Conclusions:**

Our data reveal in this Gambian population the presence of a dominant clone of *S. aureus* harbouring plasmid-encoded azithromycin resistance, which was acquired by participants in both arms of the study. Understanding these resistance dynamics is crucial to defining the public health drug resistance impacts of azithromycin prophylaxis given during labour in Africa.

## Introduction

Azithromycin, a second-generation broad-spectrum macrolide, is used to treat infections such as pneumonia, middle ear infections and sexually transmitted infections.^1^^,^^2^ It has also been used in mass drug administration (MDA) campaigns to control trachoma in several endemic countries in Africa.^3–5^ The impact of these MDA campaigns has varied from one country to another.^6–8^ MDA with azithromycin (MDA-Z) may have beneficial effects beyond trachoma control, having been shown to reduce asymptomatic pneumococcal carriage for at least 1 month,^9^ and all-cause mortality in children.^10^^,^^11^ However, a concern is that MDA-Z has been associated with an increase in the prevalence of macrolide-resistant bacterial species, even after the administration of only a single dose.^12^ The spread of these resistant bacterial populations and the associated risk to regions that may implement MDA-Z are not fully understood.

*Staphylococcus aureus* is regularly implicated as a species in which azithromycin resistance emerges following MDA-Z campaigns. In Papua New Guinea, the proportion of azithromycin-resistant (azithromycin^R^) *S. aureus* was five times higher among pregnant women treated with azithromycin than in those in the control group.^13^ A study in rural Gambia showed that three annual rounds of MDA-Z were associated with a long-term increase in the prevalence of azithromycin^R^*S. aureus*. There are three recognized types of acquired macrolide resistance mechanisms in *S. aureus*: (i) methylation of the ribosomal target (*erm* gene);^14^^,^^15^ (ii) active efflux [*msr*(A) gene];^14^^,^^16^^,^^17^ and (iii) inactivation of the macrolide (*mph*/*ere* gene).^18^^,^^19^ In the absence of these genes, mutations in ribosomal proteins have been implicated in macrolide resistance.^20^ As azithromycin is a synthetic analogue of erythromycin, it is presumed that these resistance mechanisms are active against both antimicrobials. However, molecular data confirming this association are limited for azithromycin, in particular for the efflux mechanism encoded by *msr*(A). Further, there are limited data available on the distribution of these macrolide resistance-encoding genes following public health interventions using azithromycin.

A recent MDA-Z trial in The Gambia, the Prevention of Bacterial Infections in Newborn (PregnAnZI) trial, a double-blinded placebo-controlled trial in which oral intra-partum azithromycin (2 g) was administered, showed that phenotypic resistance to azithromycin was associated with *S. aureus* isolates harbouring *msr*(A) or *erm*(C) genes.^21^ Public health interventions using antibiotics, in addition to driving the emergence of resistance, may also greatly alter the local molecular epidemiology of *S. aureus*. The distribution of dominant MSSA clones in Africa is heterogeneous,^22^ and in The Gambia there is currently a paucity of data on the prevalence of *S. aureus* STs, although ST15 and ST5 have been most commonly reported.^23^^,^^24^ The ST15 lineage in Africa has been reported to frequently harbour genes encoding the Panton–Valentine leucocidin (PVL) toxin (25.9%–90.0%) and enterotoxin A (22.0%–74.6%), suggesting a potential for increased virulence in this lineage.^22^

The recent PregnAnZI trial was undertaken to assess the efficacy of one oral dose of azithromycin administered to women during labour in lowering bacterial carriage both in the mother and in her newborn as a necessary step to reduce puerperal and neonatal sepsis. The trial revealed that azithromycin treatment significantly decreased carriage of *S. aureus*, group B *Streptococcus* and *Streptococcus pneumoniae*, but increased the prevalence of azithromycin^R^*S. aureus*, amongst the population of bacterial isolates recovered during a 28 day follow-up period.^12^ However, carriage of the latter was observed to wane in babies 12 months after delivery.^25^

The primary aims of this study were to: (i) use genomics to characterize the population of azithromycin^R^*S. aureus* recovered from mothers and babies during a 28 day follow-up period; (ii) identify the genetic mechanisms responsible for azithromycin resistance in this population and their genetic context; and (iii) explore the potential roles of clonal replacement and transmission of azithromycin^R^*S. aureus* at an individual patient level.

## Patients and methods

Additional Materials and methods are provided in the Supplementary data, available at *JAC* Online.

### PregnAnZI trial

The PregnAnZI trial was a Phase III, double-blind, placebo-controlled trial where 829 pregnant women attending the labour ward received a single oral dose (2 g) of azithromycin or placebo (ratio 1:1). The study protocol has been described elsewhere.^26^ Participants were monitored for 8 weeks and nasopharyngeal swabs were collected during the first 4 weeks of the follow-up (day 0 for mothers and days 3, 6, 14 and 28 for mothers and babies).

Trial registration (main trial): ClinicalTrials.gov Identifier NCT01800942.

### Ethics

The trial was approved by the Joint MRC/Gambia Government Ethics Committee. Mothers of children signed informed consent.

### Sample selection

**Figure 1.**
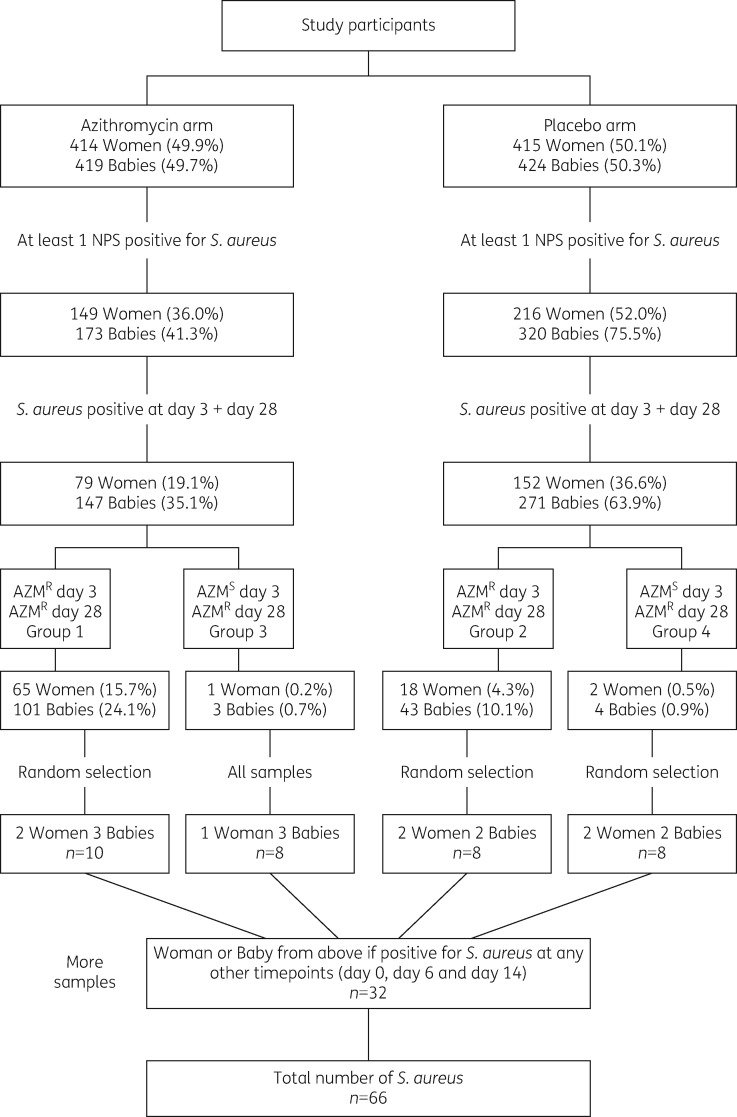
Flow chart showing the sample selection criteria from women and babies in the PregnAnZI trial. NPS, nasopharyngeal swab; AZM^R^, azithromycin resistant; AZM^S^, azithromycin susceptible. Resistance Etest cut-off values: azithromycin ≥8 mg/L.

To explore potential genetic diversity amongst azithromycin^R^*S. aureus*, we stratified isolates collected at day 3 and day 28 into four groups (Figure 1). Two groups were participants in the azithromycin treatment arm where an azithromycin^R^*S. aureus* was recovered at both timepoints (group 1) or only at day 28, with an azithromycin-susceptible (azithromycin^S^) *S. aureus* identified at day 3 (group 3). The other two groups represented the same microbiological division, but for samples recovered from participants assigned to the placebo arm [azithromycin^R^*S. aureus* at day 3 and day 28 (group 2) or azithromycin^S^*S. aureus* at day 3 and azithromycin^R^*S. aureus* at day 28 (group 4)]. A total of 17 participants (34 isolates) were selected from these four groups. In addition, all *S. aureus* recovered from the above subjects at other timepoints (days 0, 6 and 14) were included.

### WGS and bioinformatic analyses

WGS was performed on the NextSeq 500 (Illumina) using 2 × 150 bp chemistry. One isolate (S80062MN28) was subjected to long-read sequencing on the RS-II (Pacific Biosciences). Bioinformatic approaches and analyses included assembly, annotation and comparative genomics. The global *S. aureus* phylogeny was inferred using publicly available genomes. Antibiotic resistance and virulence gene detection was performed using the NCBI antimicrobial resistance database (ncbi: updated 20 September 2018) and the virulence factor database (vfdb: updated 14 August 2018). All sequence data generated for this study have been made publicly available through the European Nucleotide Archive, project accession PRJEB31151.

### Cloning and transformation of erm(C) and msr(A) genes

An empty vector control and a vector containing *erm*(C) or *msr*(A) were electroporated into an ST5 azithromycin^S^*S. aureus* strain (S70065MN00). Colonies were screened to confirm the presence of either *erm*(C) or *msr*(A) genes using PCR.

### Bacterial conjugation

Duplicate conjugation experiments were performed using an azithromycin-resistant donor strain (S80062MN28) and an azithromycin-susceptible recipient strain (S70065MN00). Resistance profiles of donor, recipient and transconjugants are described in Table S1.

### Antimicrobial susceptibility testing

Phenotypic susceptibility to azithromycin and erythromycin was determined using Etest (bioMérieux), performed as per the manufacturer’s recommendations.

### Statistics

Fisher’s exact test was used to compare the prevalence of STs or macrolide resistance genes between the azithromycin and placebo groups. A *P* value of 0.05 was used as the cut-off for statistical significance. All analyses were done using STATA/SE v12.1 (https://www.stata.com/).

## Results and discussion

### Genomic characterization of the study population

**Figure 2.**
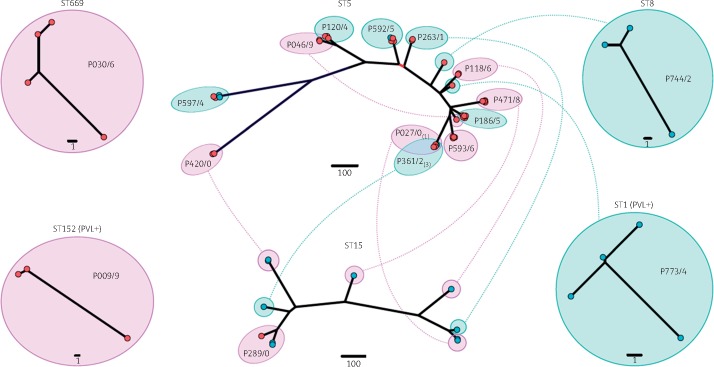
Distribution and phylogenetic relatedness of participant isolates. Illustrated are ML phylogenetic trees for each ST identified in the study population (excluding the ST1-SLV), inferred from whole-genome alignments. Branch tips are coloured based on phenotypic susceptibility to azithromycin (blue=susceptible and red=resistant, as determined by Etest). Scale bar indicates the estimated substitutions per site. Isolates recovered from the same participant are indicated by a larger circle, labelled with the participant ID and coloured based on the treatment arm to which the participant was assigned (purple=azithromycin and green=placebo). Participants with isolates representing multi-STs are connected by a dotted line. Isolates belonging to ST1 and ST152 were found to harbour the PVL toxin-encoding gene, *lukFS*.

A total of 66 *S. aureus* isolates recovered from 7 mothers and 10 babies were included in this study. Of the 66 isolates, half (*n* = 33) were recovered from the nine participants selected from the azithromycin-treatment arm and the other half from the eight participants selected from the placebo arm of the PregnAnZI trial. *In silico* MLST of the 66 *S. aureus* isolates revealed seven different STs: ST1 [and a novel ST1 single-locus variant (ST1-SLV)], ST5, ST8, ST15, ST152 and ST669 (Table 1). Overall ST5 was the dominant ST, representing 61.0% of isolates (*n* = 40) recovered from 14 participants, followed by ST15, representing 17.0% of isolates (*n* = 11) recovered from 7 participants. The prevalence of ST5 was higher in the placebo arm (73.0% versus 49.0%, *P* = 0.039), while the prevalence of ST15 was higher in the azithromycin treatment arm (27.0% versus 6.0%, *P* = 0.022). These finding are largely consistent with previous molecular epidemiological data from The Gambia, in which ST15 and ST5 were common STs identified in cases of both colonization and disease.^23^^,^^24^ Unlike previous reports of increasing PVL positivity in the Gambian ST15 population,^22^ the *lukFS* genes were only identified in the isolates representing ST1 and ST152 (Figure 2).

**Table 1.** Distribution of STs and macrolide resistance amongst the study population

	All samples		Azithromycin treatment arm		Placebo treatment arm	
MLST	all	AZM^R^	all	AZM^R^	all	AZM^R^
ST5	14 (40)	14 (36)	6 (16)	6 (16)	8 (24)	8 (20)
ST15	7 (11)	1 (1)	5 (9)	1 (1)	2 (2)	0 (0)
ST1	1 (4)	0 (0)	–	–	1 (4)	0 (0)
ST669	1 (4)	1 (4)	1 (4)	1 (4)	–	–
ST8	1 (3)	0 (0)	–	–	1 (3)	0 (0)
ST152	1 (3)	1 (3)	1 (3)	1 (3)	–	–
ST1-SLV	1 (1)	1 (1)	1 (1)	1 (1)	–	–

AZM^R^, azithromycin resistant. Data are shown as number of participants (number of isolates).

**Figure 3.**
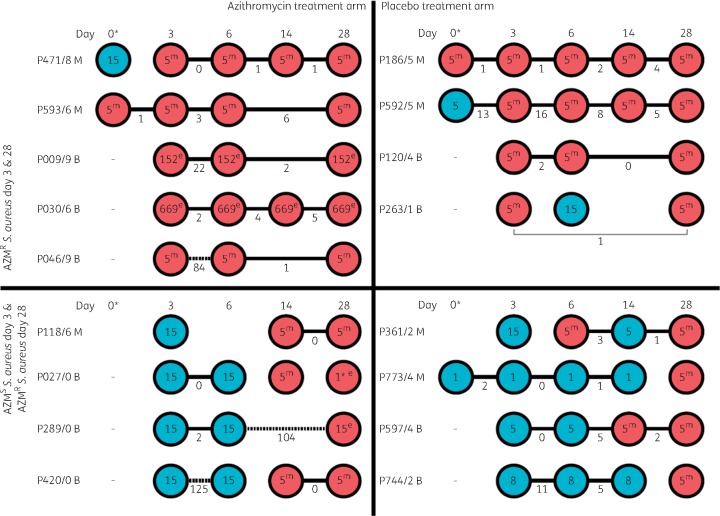
Individual participant timelines. Illustrated are the isolate timelines for each participant selected for the study, grouped based on the selection criteria outlined in Figure 1. Each participant is indicated by their participant ID and the adjacent circles represent the isolates recovered from samples taken prior to the intervention (day 0 for mothers) and following the intervention on days 3, 6, 14 and 28 (for mothers and babies). Isolate circles are coloured based on the phenotypic susceptibility to azithromycin (blue=susceptible and red=resistant, as determined by Etest). The internal number indicates the *in silico* MLST of the isolate and the superscript letter indicates the macrolide resistance gene detected, ‘m’ for *msr*(A) and ‘e’ for *erm*(C). Black lines connecting adjacent isolates indicate that the same clone has probably been maintained, with the pairwise core SNP distance provided. Alternatively, gaps between adjacent isolates indicate a potential clonal replacement event. AZM^R^, azithromycin resistant; AZM^S^, azithromycin susceptible.

To explore the genetic relationships between isolates, a maximum likelihood (ML) phylogenetic tree was inferred for each ST (excluding the ST1-SLV). In the case of ST1, ST8, ST152 and ST669, each clone was recovered from a single participant and demonstrated limited core SNP diversity (Figure 2). This finding suggested that these participants maintained the same *S. aureus* clone over the 28 day follow-up period. Only ST5 and ST15 were recovered from multiple participants (*n* = 14 and 7, respectively) and as a group demonstrated core SNP diversity. Isolates of the same ST recovered from the same participant, however, largely formed a single, genetically distinct clade within both phylogenetic trees (Figure 2). The median pairwise core SNP distance between isolates recovered from the same participant was 2.5 (±16.4, range 0–85) for ST5 and 104 (±61.2, range 0–125) for ST15; for isolates recovered from different participants it was 95 (±34.2, range 0–200) for ST5 and 115 (±24.1, range 9–136) for ST15. A potential clonal replacement event probably occurred in one participant (P046/9), with the ST5 MSSA isolate recovered on day 3 having a pairwise core SNP distance of 84 and 85 to the ST5 MSSA isolates recovered on day 6 and 28, respectively (Figure 3, Dataset S3). Another two participants (P027/0 and P0361/2) appeared to be involved in a potential transmission event as the four ST5 MSSA isolates recovered from these participants demonstrated a pairwise core SNP distance of ≤3 (Dataset S3). There was no apparent clustering of participant isolates based on the treatment received (Figure 2).

### Genetic basis of azithromycin resistance

Of the 66 *S. aureus* isolates, 21 were phenotypically azithromycin^S^ and 45 azithromycin^R^ as determined by Etest. None of the azithromycin^S^*S. aureus* carried a known azithromycin resistance-conferring gene, whereas all azithromycin^R^*S. aureus* were found to carry either an *erm*(C) (*n* = 9, 20%) or *msr*(A) (*n* = 36, 80%) gene (Figure 3), genes associated with macrolide resistance. Mutations in genes encoding ribosomal proteins (*rplD*, *rplV* and 23S rRNA) were investigated. Only two missense mutations were identified: RplD A_133_D and RplD T_145_I. Neither has been previously reported, and they were identified in four isolates carrying *erm*(C) (P471/8) and one isolate carrying *msr*(A) (P027/0), respectively.

The two macrolide resistance genes are associated with different phenotypes: carriage of *msr*(A) among staphylococci is associated with phenotypic resistance to 14-membered (clarithromycin, dirithromycin and erythromycin) or 15-membered (azithromycin) ring macrolides and streptogramin A, but susceptibility to 16-membered ring macrolides.^18^ Carriage of *erm* genes in staphylococci is associated with a broader phenotypic resistance depending on whether the gene is inducible or constitutively expressed. The former, often referred to as iMLS_B_, mediates resistance to 14- and 15-membered macrolides and streptogramin B, but susceptibility to 16-membered macrolides and lincosamides, with a risk of constitutive expression arising *in vivo*.^18^^,^^27^ The latter, denoted by cMLS_B_, mediates resistance to all macrolide, lincosamide and streptogramin B antibiotics.^18^

The prevalence of each gene differed significantly when isolates were grouped based on the intervention which the participant received (*P* = 0.002). The *erm*(C) gene was carried by isolates belonging to ST15, ST152, ST669 and the ST1-SLV, and were only recovered from participants assigned to the azithromycin treatment arm. Conversely, the *msr*(A) gene was exclusively associated with ST5 MSSA isolates and was recovered from participants assigned to either treatment arm (Figure 3). As both resistance genes would provide protection against azithromycin, the factors mediating the differences in the prevalence of each gene between participants assigned to the different interventions are unclear. This finding requires confirmation in a larger isolate dataset.

### Genetic context of msr(A)

**Figure 4.**
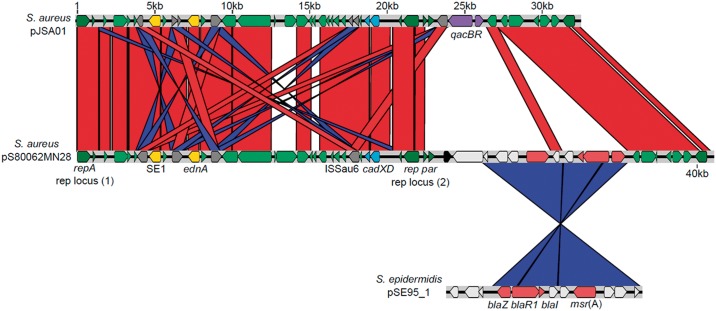
Schematic for the structure of pS80062MN28. Illustrated is the structure of pS80062MN28 (central sequence PRJEB31151) with genes of interest annotated. pS800262MN28 has been aligned with *S. aureus* plasmid pJSA01 (accession AP014922.1) and *S. epidermidis* plasmid pSE95_1 (accession CP024438.1). Connecting lines indicate sequences that share >99% nucleotide sequence homology, coloured red or blue if the sequence was present in the same or reverse orientation, respectively.

In the ST5 lineage identified in this study, *msr*(A) was found to be located on an MDR staphylococcal plasmid (Figure 4). pS80062MN28 is 41069 bp in length and demonstrates significant similarity to the published *S. aureus* plasmid pJSA01 (accession AP014922.1) (Figure 4).^28^ Plasmid pJSA01 carries two virulence genes, a newly discovered enterotoxin (SE1) and the epidermal cell differentiation inhibitor A (EDIN-A) encoded by *ednA*.^28^^,^^29^ Additionally, it carries genes encoding cadmium resistance (*cadXD*) and biocide tolerance (*qacBR*).^28^ Plasmid pS80062MN28 was found to carry both putative virulence genes and *cadXD*, but not *qacBR*, and contained a 12 kb region not present in pJSA01 (Figure 4). Approximately 9 kb of this region demonstrated significant similarity to a putative *S. epidermidis* plasmid pSE95_1 (accession CP024438.1),^30^ and included *msr*(A) and the β-lactamase resistance-encoding gene *blaZ* (Figure 4). In all *msr*(A)-harbouring ST5 MSSA isolates, an ∼27 kb contig was identified, representing the plasmid region that spans from the ISSau6-type transposase upstream of *cadXD* to another copy upstream of SE1 (Figure 4), suggesting that all isolates probably carried a pS80062MN28-like plasmid.

To demonstrate that *msr*(A) was responsible for macrolide resistance in the ST5 lineage, a copy of the gene was transformed into a macrolide-susceptible ST5 isolate from the study collection (S70065MN00), using vector pRAB11.^31^ Two allelic variants of *erm*(C) [*erm*(C)*_2* and *erm*(C)*_13*] were also tested. Transformation of an empty vector did not result in a change in azithromycin or erythromycin susceptibility in isolate S70065MN00; however, transformation of any one of the three resistance genes resulted in an increase in the azithromycin MIC from 1.0 to >256 mg/L (Figure S1), the first time introduction of *msr*(A) or *erm*(C) genes in a previously azithromycin^S^*S. aureus* clinical isolate has been shown to result in azithromycin resistance. All clinical *S. aureus* isolates carrying either the *erm*(C) or *msr*(A) gene had similarly high MIC values as those shown in the cloning experiment (MIC ≥256 mg/L). The MIC values for all azithromycin-resistant and -susceptible isolates are included in Dataset S1 (column F). WGS of transformants demonstrated no additional mutations potentially contributing to azithromycin resistance. Therefore, acquisition of *msr*(A), probably through uptake of the plasmid, is responsible for macrolide resistance in the ST5 lineage. Results of the bacterial conjugation experiment demonstrated that the transfer of the *msr*(A)-containing resistance plasmid was <1.06 × 10^−14^ between *S. aureus*, indicating a low frequency of transmission.

### Contextualization of the study population

**Figure 5.**
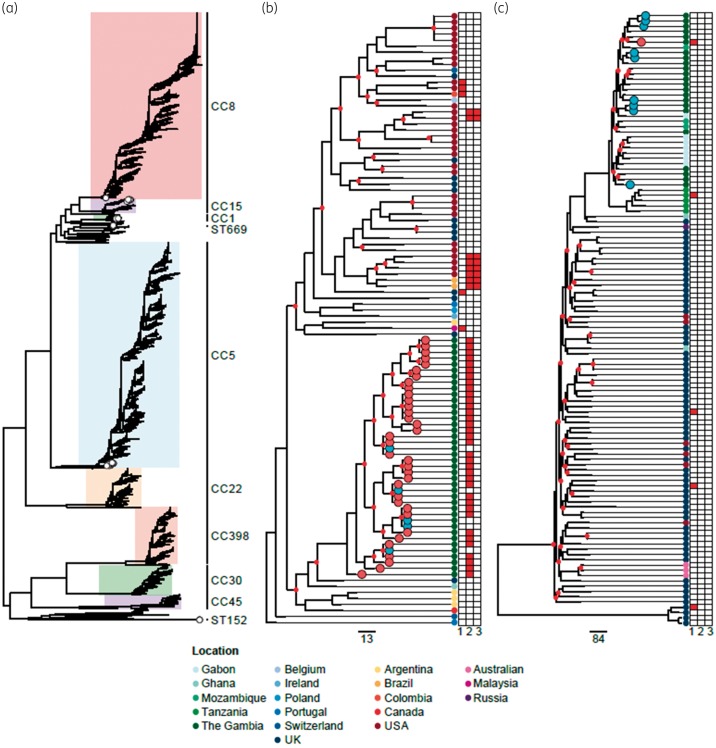
Phylogenetic contextualization of study isolates. (a) Illustrated is an ML phylogenetic tree for a global population of 7192 *S. aureus* isolates. The Gambian isolates identified in this study are indicated by white circles located at the branch tips. (b) A subtree of CC5 isolates and (c) a subtree of CC15 isolates. In both subtrees, branches with <85% support (approximate likelihood ratio test) are labelled with a red circle. The Gambian isolates identified in this study are indicated by circles located at the branch tips, coloured based on phenotypic susceptibility to azithromycin (blue=susceptible and red=resistant, as determined by Etest). Adjacent to each subtree is a vertical line of circles indicating the countries from which the isolates were recovered (refer to key); the heatmap indicates the presence or absence of three macrolide resistance genes: *erm*(C) (1), *msr*(A) (2) and *mph/ere* (3). The scale bar indicates the estimated number of core genome substitutions.

To contextualize the Gambian isolates of this study with the global population of *S. aureus*, an ML phylogenetic tree comprising 7126 publicly available *S. aureus* genomes together with the sequenced isolates of this study was constructed, as well as subtrees of CC5 and CC15 (Figure 5). The subtree of CC5 isolates (Figure 5b) illustrated that the Gambian ST5 MSSA lineage identified in this study represented a single monophyletic clade, consistent with local expansion of this clone. The most closely related isolate to this clade was recovered from the UK (435 SNPs). Given the distribution of macrolide resistance genes in this CC5 population (Figure 5b), there is no clear source for pS80062MN28 in the Gambian ST5 lineage. Therefore, it remains unclear if the MDA-Z trial promoted the expansion of this lineage (already carrying the plasmid) or provided the necessary selection pressure for plasmid uptake from a currently unidentified source. The potential consequences of either evolutionary mechanism are greater than simply increasing the prevalence of azithromycin resistance in a region due to the co-location of *msr*(A) with other resistance and virulence genes on the plasmid. Therefore, the impact of pS80062MN28 carriage for bacterial fitness and pathogenicity requires further investigation.

### Population dynamics of azithromycin^R^ S. aureus carriage

As all participants were sampled at multiple and consistent timepoints following administration of either azithromycin or the placebo, it presented an opportunity to explore the *S. aureus* population dynamics of each participant during the 28 day follow-up period. The isolate timelines are illustrated in Figure 3. Amongst the 17 participants, two common patterns were observed and reflected the way in which isolates had been selected. First, if a participant carried an azithromycin^R^*S. aureus* at day 0 or day 3 (representing early/pre-intervention acquisition), the same clone was identified at day 28, suggesting that it had been maintained regardless of ST or the macrolide resistance gene identified during the 4 weeks post-intervention. This pattern was observed in all nine participants in groups 1 and 2 (Figure 3). Second, if a participant acquired an azithromycin^R^*S. aureus* after day 3 (representing delayed acquisition), then the first azithromycin^R^ clone acquired was an ST5 MSSA harbouring *msr*(A). This pattern was observed in seven of the eight participants in groups 3 and 4 (Figure 3). Further, this was also observed in the two mothers from groups 1 and 2 in which an azithromycin^S^*S. aureus* was recovered at day 0 (pre-intervention) and an azithromycin^R^*S. aureus* at day 3 (Figure 3, P593/6 and P592/5). In two participants, it appeared that the azithromycin^R^ ST5 isolate recovered at day 6 was replaced by either an azithromycin^S^ ST5 (P361/2) or an azithromycin^S^ ST15 (P263/1) isolate at day 14, which then switched back to the azithromycin^R^ ST5 isolate at day 28 (Figure 3). This could represent repeated clonal replacement in these participants, dual carriage of both clones (which would be missed by this study as only a single colony was sequenced from each sample) or, in the case of P361/2, *in vitro* plasmid loss. It is also unclear why the four participants in group 3 only carry azithromycin^S^ ST15 MSSA prior to acquiring azithromycin^R^ MSSA, whereas group 4 are more varied (Figure 3). Again, this is possibly due to the small sample size considered in this study or may reflect an unknown selection pressure promoting short-term carriage of the azithromycin^S^ ST15 clone in participants assigned to the azithromycin treatment arm.

Collectively these isolate timelines suggest that participants who received the azithromycin intervention either maintained the azithromycin^R^ MSSA, which they probably carried prior to the intervention, or acquired one, replacing an azithromycin^S^ ST15 MSSA, with the resulting azithromycin^R^ MSSA being of varied ST and carrying either *msr*(A) or *erm*(C). In participants who received the placebo, only azithromycin^R^ ST5 MSSA was acquired, replacing various azithromycin^S^ MSSA populations. There are multiple potential explanations for this finding. The increased use of azithromycin may have significantly altered the local molecular epidemiology of *S. aureus*, favouring azithromycin^R^ clones. When combined with widespread transmission, this could explain the high prevalence of the ST5 lineage if it additionally has a colonization advantage over other azithromycin^R^ clones. The absence of this lineage in the babies sampled at 1 year post-intervention suggests that any colonization advantage, if indeed present, is only advantageous in the presence of high azithromycin use. Without a snapshot of the molecular epidemiology of *S. aureus* in the catchment area prior to the trial, limited conclusions can be drawn about the impact that the trial has had on the *S. aureus* population and highlights the need for such information prior to any subsequent MDA campaigns.

### Conclusions

We have used comprehensive genomic analyses to reveal the dynamics of azithromycin^R^*S. aureus* colonization in mothers and babies after azithromycin treatment or placebo. Plasmid-encoded *msr*(A) in ST5 MSSA was the most common clone, being responsible for most azithromycin^R^*S. aureus* acquisitions in both study arms. These results provide critical information to inform a greater understanding of the ecological impact of azithromycin prophylaxis on staphylococcal populations in Western Africa.

## Supplementary Material

dkz341_Supplementary_Data
