# Supplementary material for: Genomic investigation of Staphylococcus aureus recovered from Gambian women and newborns following an oral dose of intra-partum azithromycin
Source: J Antimicrob Chemother. 2019 Aug 19;74(11):3170–8. doi: 10.1093/jac/dkz341 (PMC6798832; doi:10.1093/jac/dkz341)
Supplement: dkz341_Supplementary_Data [file dkz341_supplementary_data.zip › Supplementary_data.docx]

**Supplementary data**

**Supplementary Materials and Methods**

**PregnAnZI trial**

The PregnAnZI trial recruited women delivering at the Bundung Maternal and Child Health Hospital (BMCHH) formerly called Jammeh Foundation for Peace (JFP), a government-run health facility located in Western Gambia that manages on average 4,500 deliveries per year. The population of the catchment area of (BMCHH) includes the main ethnic groups in The Gambia and the illiteracy rate is high. Details of the study protocol have been described elsewhere [[1](#_ENREF_1)]. In brief, it was a phase-III, double-blind, placebo-controlled trial where 829 pregnant women attending the labour ward in the study health facility were randomized to receive a single oral dose of 2g of either oral AZI or a placebo (ratio 1:1). Recruitment of trial participants started in April 2013 and lasted 12 months, with an additional 2 months of follow-up. Study participants were monitored during eight weeks following the intervention and nasopharyngeal swabs (NPS) where collected during the first 4 weeks of the follow-up (day 0 for mothers, and day 3, 6, 14, and 28 for mothers and babies). Other biological samples were also collected during the follow-up period but were not considered for this genomic study [[2](#_ENREF_2)]. All study mothers had signed consent during their pre-natal visits before being enrolled into the trial. The trial was approved by the Joint MRC/Gambia Government Ethics Committee.

**Sample selection**

In order to explore the potential genetic diversity amongst AZI^R^ *S. aureus* recovered during the trial, we stratified isolates collected at day 3 and day 28 into four groups (Figure 1). Two groups represented samples from participants assigned to the AZI treatment arm where an AZI^R^ *S. aureus* was recovered at both time points (Group 1) or only at day 28, with an AZI susceptible (AZI^S^) *S. aureus* identified at day 3 (Group 3). The other two groups represented the same microbiological division but for samples recovered from participants assigned to the placebo arm (AZI^R^ *S. aureus* at day 3 and day 28 [Group2], or AZI^S^ *S. aureus* at day 3 and AZI^R^ *S. aureus* at day 28 [Group 4]). A total of 17 participants (34 isolates) were selected from these four groups for this genomic study. To further explore the population dynamics of AZI^R^ *S. aureus* carriage over the follow-up period, all *S. aureus* recovered from the above subjects at other time points (days 0, 6 and 14) were also included. Further detail about isolate selection is provided in Figure 1.

**Whole Genome Sequencing**

Genomic DNA was extracted using the GenElute^TM^ Bacterial Genomic DNA Kit (Sigma). DNA libraries were prepared using the Nextera XT kit (Illumina) and sequenced on the NextSeq 500 (Illumina) using 2 × 150-bp chemistry. One isolate (S80062MN28) was subjected to long-read sequencing on the RS-II (Pacific Biosciences) using P6-C4 chemistry. All sequence data generated for this study has been made publicly available through the European Nucleotide Archive, project accession PRJEB31151.

**Bioinformatic Analysis**

Short-read sequence data was assembled using SPAdes v3.11.1 [[3](#_ENREF_3)], and contigs having a coverage less than 5 were excluded as contaminants. Isolate S80062MN28 was assembled from both long and short read sequence with Unicycler v0.4.6 in “bold” mode [[4](#_ENREF_4)]. Annotation of plasmid pS80062MN28 was performed using Prokka v1.13.3[[5](#_ENREF_5)], using plasmid pJSA01 (accession AP014922.1) [[6](#_ENREF_6)]. Comparative alignment of plasmid sequences was performed using the Artemis Comparison Tool v17.0.1, excluding matches ≤ 100 bp in length[[7](#_ENREF_7)]. *In silico* MLST and antibiotic resistance gene detection was performed on draft assemblies and complete genomes using mlst v2.10 (<https://github.com/tseemann/mlst>) and abricate v0.8.10 (<https://github.com/tseemann/abricate>), using the NCBI antimicrobial resistance database (retrieved 20-Sept-2018) and the virulence factor database (retrieved 14-Aug-2018).

For the phylogenetic analysis, short-read sequence data was aligned to the complete genomes of six *S. aureus* reference genomes (genome accessions: CP026964.1 [ST1]; CP026968.1 [ST5]; CP012970.1 [ST8]; LS483319.1 [ST15]; CP024998.1 [ST152]; LS483314.1 [ST669]), each selected for being the most closely related complete genome for each ST identified in the study population. Variant calling was achieved using Snippy v4.3.5 (<https://github.com/tseemann/snippy>). Whole genome alignments were used to construct maximum likelihood (ML) phylogenetic trees with IQtree v1.6.5 [[8](#_ENREF_8)]. Model selection was used to find the best nucleotide substitution model for each alignment (chosen according to the Bayesian Information Criterion): this was either the HKY+F for ST5 and ST15 [[9](#_ENREF_9)], or the F81+F for ST1, ST8, ST152 and ST669 [[10](#_ENREF_10)]. Alignments composed of greater than 5 isolates (ST5 and ST15) were subjected to 1000 ultra-fast bootstrap replicates [[11](#_ENREF_11)]. ML trees were visualized using FigTree v1.4.3 (http://tree.bio.ed.ac.uk/software/figtree/). Core pairwise SNP distances were calculated using an in-house pearl script.

The global *S. aureus* phylogeny was constructed using publicly available genomes and metadata as described in Guerillot *et al* [[12](#_ENREF_12)]. The 66 isolates sequenced as part of this study were added, together with 28 newly sequenced *S. aureus* genomes from Africa (downloaded from the Staphopia database[[13](#_ENREF_13)]. The 7191 genomes were mapped to the fully assembled reference genome *S. aureus* NRS384 and core genome alignment of the global collection (n=7192), all CC5 isolates (n=2735) or all CC15 isolates (n=179) were generated with snippy v4.3.6. Maximum likelihood phylogenetic trees were inferred with FastTree v2.1.8 using the generalized time-reversible (GTR) model. Trees were plotted and annotated using the R library *ggtree*[[14](#_ENREF_14)]. Sub-trees of CC5 and CC15 were generated from the full CC5 or CC15 phylogenetic trees by extracting only isolates with a tip distance of < 23 nodes from the isolates sequenced in this study.

Detection of mutations in ribosomal proteins (*rplD*, *rplV*, and 23S rRNA) were performed using Snippy v4.3.8. Because of the multi-copy nature of the 23S rRNA, mutations were assessed by mapping short-read sequence data to a single copy of the gene from CP026968.1 [ST5] and calling mutations at a minimum mapped-base frequency of 0.5 (option “--min-frac 0.5”). Mutations were not called at a lower frequency as it was expected that they would need to be present in the majority of 23S rRNA copies to impact of phenotypic resistance[[15](#_ENREF_15), [16](#_ENREF_16)].

**Cloning and transformation of *erm*C and *msr(A)* genes**

Vector pRAB11 was initially amplified using primers pRAB11_F and pRAB11_R to create a backbone for SLiCE (seamless ligation cloning extract) cloning (all primer sequences are provided in Supplementary Dataset 2)[[17](#_ENREF_17)]. The *erm*C and *msr(A)* genes were then amplified using primers *erm*C_pRAB11_F and *erm*C_pRAB11_R, and *msr(A)*_Saur_pRAB_F and *msr(A)*_Saur_pRAB_R, respectively. The PCR fragments were ligated using SLiCE and transformed into IM08B competent *E. coli* cells[[18](#_ENREF_18)] . The transformed cells were plated on Luria broth (LB) agar with 100µg/ml ampicillin and incubated overnight at 37ºC. Successful transformation was confirmed by colony PCR. The confirmed colonies were cultured overnight in LB with 100µg/ml ampicillin and plasmids extracted using QIAGEN mini prep kit. The empty vector or those containing *erm*C or *msr(A)* were electroporated into an ST5 AZI^S^ *S. aureus* strain (S70065MN00) and plated on LB agar with 10µg/ml chloramphenicol before incubating overnight. Colonies were screen to confirm the presence of either *ermC* or *msr(A)* genes using the above primers. Antimicrobial susceptibility was tested as outlined below.

**Bacterial conjugation**

The donor (S80062MN28; azithromycin MIC 192 μg/ml, rifampicin MIC <1 μg/ml; fusidic acid MIC <1 μg/ml) and recipient strain that was induced to high level rifampicin and fusidic acid resistance through serial passage (S70065MN00, azithromycin MIC 1 μg/ml, rifampicin MIC >32 μg/ml; fusidic acid MIC >32 μg/ml) were mixed on a membrane filter (Millipore) with a donor-to-recipient ratio of 1:1. Plates were incubated overnight at 37°C. The bacterial cells were resuspended in PBS and spread on BHI agar plates containing the appropriate antibiotics. To quantify conjugative transfer efficiencies, dilutions were plated on BHI media containing antibiotics for the selection of; donor cells, recipient cells and plasmid containing recipient cells. Transconjugants were selected on plates containing azithromycin, rifampicin and fusidic acid all at 32 μg/ml. All conjugation assays were performed in duplicate. Control conjugation experiments were performed with the donor strain only, and no spontaneous resistant colonies were observed. The resistance profile of donor, recipient and transconjugant are summarised in Table S1.

**Antimicrobial susceptibility testing**

Phenotypic susceptibility to AZI and erythromycin was determined using Etest (BioMérieux), performed as per the manufacturer’s recommendations. Etest values < 4µg/ml were interpreted as sensitive , ≥ 8 μg/ml as resistant, and ≥ 4 μg/ml but < 8 μg/ml as intermediate, as per the CLSI guidelines [[19](#_ENREF_19)].

**Statistics**

Fisher’s exact test was used to compare the prevalence of sequence types or macrolide resistance genes between the azithromycin and placebo group. A p value of 0.05 was used as the cutoff for statistical significance. All analyses were done using STATA/SE v12.1 (<https://www.stata.com/>).

**References**

1. Roca A, Oluwalana C, Camara B, Bojang A, Burr S, Davis TM, Bailey R, Kampmann B, Mueller J, Bottomley C *et al*: **Prevention of bacterial infections in the newborn by pre-delivery administration of azithromycin: Study protocol of a randomized efficacy trial**. *BMC pregnancy and childbirth* 2015, **15**:302.

2. Roca A, Oluwalana C, Bojang A, Camara B, Kampmann B, Bailey R, Demba A, Bottomley C, D'Alessandro U: **Oral azithromycin given during labour decreases bacterial carriage in the mothers and their offspring: a double-blind randomized trial**. *Clinical microbiology and infection : the official publication of the European Society of Clinical Microbiology and Infectious Diseases* 2016, **22**(6):565 e561-569.

3. Bankevich A, Nurk S, Antipov D, Gurevich AA, Dvorkin M, Kulikov AS, Lesin VM, Nikolenko SI, Pham S, Prjibelski AD *et al*: **SPAdes: a new genome assembly algorithm and its applications to single-cell sequencing**. *Journal of computational biology : a journal of computational molecular cell biology* 2012, **19**(5):455-477.

4. Wick RR, Judd LM, Gorrie CL, Holt KE: **Unicycler: Resolving bacterial genome assemblies from short and long sequencing reads**. *PLoS computational biology* 2017, **13**(6):e1005595.

5. Seemann T: **Prokka: rapid prokaryotic genome annotation**. *Bioinformatics* 2014, **30**(14):2068-2069.

6. Hisatsune J, Hagiya H, Shiota S, Sugai M: **Complete Genome Sequence of Systemically Disseminated Sequence Type 8 Staphylococcal Cassette Chromosome mec Type IVl Community-Acquired Methicillin-Resistant Staphylococcus aureus**. *Genome announcements* 2017, **5**(35).

7. Carver TJ, Rutherford KM, Berriman M, Rajandream MA, Barrell BG, Parkhill J: **ACT: the Artemis Comparison Tool**. *Bioinformatics* 2005, **21**(16):3422-3423.

8. Nguyen LT, Schmidt HA, von Haeseler A, Minh BQ: **IQ-TREE: a fast and effective stochastic algorithm for estimating maximum-likelihood phylogenies**. *Molecular biology and evolution* 2015, **32**(1):268-274.

9. Felsenstein J: **Evolutionary trees from DNA sequences: a maximum likelihood approach**. *Journal of molecular evolution* 1981, **17**(6):368-376.

10. Hasegawa M, Kishino H, Yano T: **Dating of the human-ape splitting by a molecular clock of mitochondrial DNA**. *Journal of molecular evolution* 1985, **22**(2):160-174.

11. Hoang DT, Chernomor O, von Haeseler A, Minh BQ, Vinh LS: **UFBoot2: Improving the Ultrafast Bootstrap Approximation**. *Molecular biology and evolution* 2018, **35**(2):518-522.

12. Guerillot R, Goncalves da Silva A, Monk I, Giulieri S, Tomita T, Alison E, Porter J, Pidot S, Gao W, Peleg AY *et al*: **Convergent Evolution Driven by Rifampin Exacerbates the Global Burden of Drug-Resistant Staphylococcus aureus**. *mSphere* 2018, **3**(1).

13. Petit RA, 3rd, Read TD: **Staphylococcus aureus viewed from the perspective of 40,000+ genomes**. *PeerJ* 2018, **6**:e5261.

14. Yu G, Lam TT, Zhu H, Guan Y: **Two Methods for Mapping and Visualizing Associated Data on Phylogeny Using Ggtree**. *Molecular biology and evolution* 2018, **35**(12):3041-3043.

15. Sharkey LK, Edwards TA, O'Neill AJ: **ABC-F Proteins Mediate Antibiotic Resistance through Ribosomal Protection**. *mBio* 2016, **7**(2):e01975.

16. Silver LL: **Challenges of antibacterial discovery**. *Clinical microbiology reviews* 2011, **24**(1):71-109.

17. Zhang Y, Werling U, Edelmann W: **Seamless Ligation Cloning Extract (SLiCE) cloning method**. *Methods in molecular biology* 2014, **1116**:235-244.

18. Monk IR, Tree JJ, Howden BP, Stinear TP, Foster TJ: **Complete Bypass of Restriction Systems for Major Staphylococcus aureus Lineages**. *mBio* 2015, **6**(3):e00308-00315.

19. Institute CaLS: **Performance Standards for Antimicrobial Susceptibility testing**. 2014, **M100**(S24).

**Table S1.** Resistance profile of strains used in conjugation experiments

| **Strain** | **Azithromycin MIC**  μg/ml | **Rifampicin MIC** μg/ml | **Fusidic acid MIC** μg/ml |
| --- | --- | --- | --- |
| Donor (S80062MN28) | 192 | <1 | <1 |
| Recipient^#^ | 1 | >32 | >32 |
| Transconjugants | >32 | >32 | >32 |

**^#^Note.** Strain S70065MN00 induced to high level rifampicin and fusidic acid resistance through serial passage.


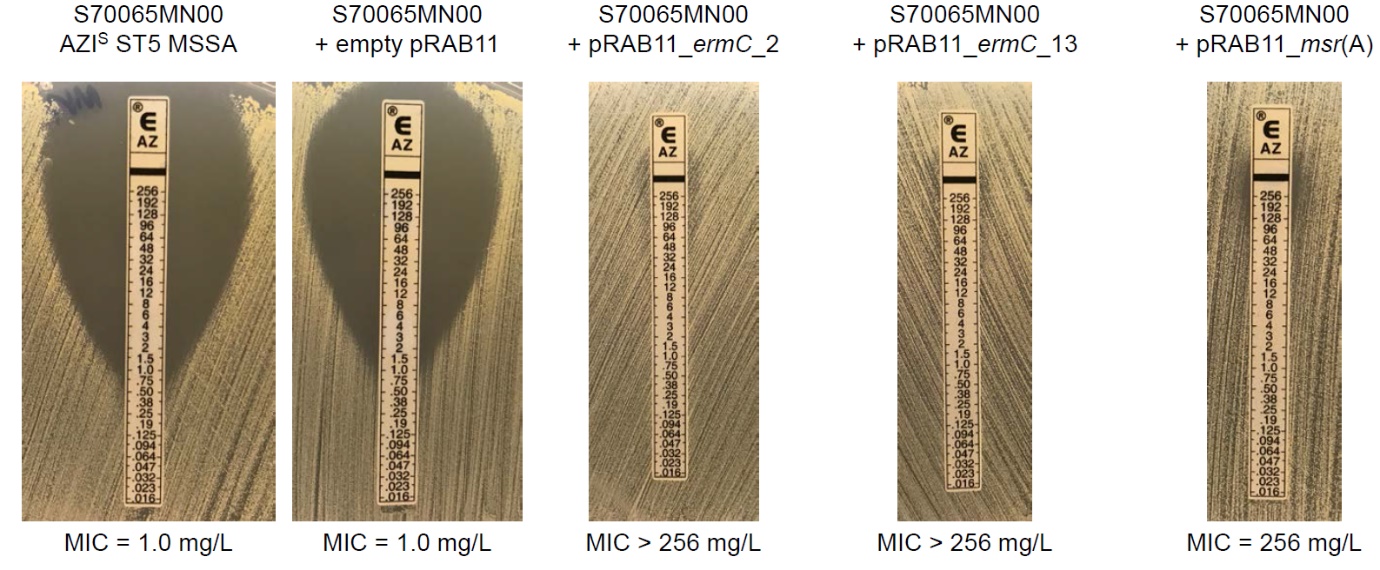


**Figure S1.** Phenotypic tests confirming that the acquisition of *ermC*_2, *ermC*_13 or *msr(A)* into a susceptible ST5 MSSA (representative of The Gambian lineage identified in this study) results in a significant increase in the minimum inhibitory concentration (MIC) to azithromycin.
